# Building a doctor, one skill at a time: Rethinking clinical training through a new skills-based feedback modality

**DOI:** 10.1007/s40037-021-00666-9

**Published:** 2021-05-26

**Authors:** Brandon Kappy, Lisa E. Herrmann, Daniel J. Schumacher, Angela M. Statile

**Affiliations:** 1 Department of Pediatrics, Division of Emergency Medicine, Cincinnati Children’s Hospital Medical Center, University of Cincinnati College of Medicine, Cincinnati, OH USA; 2 Department of Pediatrics, Division of Hospital Medicine, Cincinnati Children’s Hospital Medical Center, University of Cincinnati College of Medicine, Cincinnati, OH USA

**Keywords:** Entrustable professional activities, Milestones, Competency-based medical education, Microskills

## Abstract

The Accreditation Council for Graduate Medical Education milestones and entrustable professional activities (EPAs) are important assessment approaches but may lack specificity for learners seeking improvement through daily feedback. As in other professions, clinicians grow best when they engage in deliberate practice of well-defined skills in familiar contexts. This growth is augmented by specific, actionable coaching from supervisors. This article proposes a new feedback modality called microskills, which are derived from the psychology, negotiation, and business literature, and are unique in their ability to elicit targeted feedback for trainee development. These microskills are grounded in both clinical and situational contexts, thereby mirroring learners’ cognitive schemas and allowing for more natural skill selection and adoption. When taken as a whole, microskills are granular actions that map to larger milestones, competencies, and EPAs. This article outlines the theoretical justification for this new skills-based feedback modality, the methodology behind the creation of clinical microskills, and provides a worked example of microskills for a pediatric resident on a hospital medicine rotation. Ultimately, microskills have the potential to complement milestones and EPAs and inform feedback that is specific, actionable, and relevant to medical learners.

**Supplementary Information:**

The online version of this article (10.1007/s40037-021-00666-9) contains supplementary material, which is available to authorized users.

## Introduction

Recent advances in resident assessment have included the introduction of the Accreditation Council for Graduate Medical Education (ACGME) milestones in the United States and a rise in popularity of entrustable professional activities (EPAs) internationally [1–4]. Milestones provide narrative descriptions of steps in development across several competency domains [5, 6]. They consist of both an *analytic *framework, in which domains of competencies are hierarchically broken down into further sub-categories [7], as well as a *developmental* component, where learners progress along competency stages from novice to expert [8]. While this framing can provide learners and supervisors with a developmental roadmap, milestones are context-independent, leaving a gap in their utility [9]. EPAs fill this gap, mapping competencies and their respective milestones to core activities of a profession. EPAs “offer a method of assessment [that asks whether] the learner [is] ready to be entrusted to perform [this task] without supervision” [2]. They are *synthetic* in nature, with each activity requiring incorporation of multiple competencies to support the function of performing the EPA.

While milestones and EPAs complement one another by viewing performance through both a narrow (milestones) and wide-angle (EPAs) lens, neither places direct focus on granularity that can provide learners with actionable feedback to drive day-to-day improvement. This can present challenges for assessors using these frameworks to give targeted feedback or observe specific actions in the clinical setting [10–12]. To augment the assessment approaches enabled by milestones and EPAs with a feedback approach that is granular and specific, we propose a new modality for medical trainee assessment and feedback called microskills. Building upon the concept of deliberate practice, microskills deconstruct milestones and EPAs into a skills-based framework. By mirroring the real-world contexts in which learners practice medicine, microskills offer the opportunity to reimagine how residents engage in daily improvements, providing a supplementary method for programs to consider trainee clinical progression and deliver feedback.

## Theoretical basis and justification for microskills development

### Microskills and deliberate practice

Microskills are derived from the psychology [13, 14], negotiation [15–17], and business [18] literature and are unique in their ability to elicit targeted, relevant feedback to promote trainee development. Introduced 50 years ago, microskills (called microcounseling in psychology) transitioned the mental health education field from a theoretical to a skills-based framework. Microskills are specific and isolated actions (e.g., summarizing content back to the patient) that can be observed and repeatedly practiced by counselors, distilling complex psychological principles into understandable and repeatable skills [19]. The microskills framework remains widely used in psychology graduate programs and is the basis for numerous textbooks [20].

Decades of studies have confirmed the effectiveness of the microskills framework. Microskills-trained counselors have demonstrated improved basic and higher-level counseling skills (e.g., asking open-ended questions), established superior therapeutic alliances with patients (measured through patient satisfaction surveys), and displayed empathy better [14, 21]. These findings are summarized in several meta-analyses reviewing microskills effectiveness compared to other psychological training approaches, demonstrating statistically significant effect sizes of microskills across multiple dimensions (i.e., microcounseling is effective for teaching a variety of therapeutic communication skills) [21, 22].

The microskills framework of distilling processes into discrete, repeatable actions has been applied to other fields of study. Microskills were efficacious in teaching children problem-solving skills, adolescents interpersonal skills, and couples relationship skills [21]. Negotiation experts have distilled complicated tactics into microskills that individuals can practice, becoming a primary method for teaching negotiation today.

The idea of repeatedly engaging with skills is not new to medicine; studies have shown that residents learn and grow best when they engage in deliberate practice of highly targeted skills [23, 24]. Deliberate practice involves the attainment of expertise through (1) repeating discrete, well-defined tasks, (2) receiving immediate feedback on actions, and (3) re-engaging with the task with an intent and ideas to improve [25]. Tasks need to be specific enough so that on repeat attempts trainees can consistently improve small aspects of their performance without becoming confused by other tasks or larger goals. Each task must also be easily observable to supervisors, who can then provide targeted feedback.

To date, efforts at breaking down larger clinical functions into skills have involved trainees performing iterative and quantifiable tasks, such as reading electrocardiograms or practicing surgical skills on simulators [25, 26]. These examples focus on procedural skills in controllable circumstances with measurable outcomes (e.g., gaining speed with knot-tying). Few studies have explored deliberate practice in less controllable domains of medical training, such as patient presentations or clinical management, which involve multiple skills and cannot be easily reproduced. Perhaps the closest analogue to microskills in medicine is clinical communication skills training (CST), which teaches medical professionals verbal and nonverbal skills (i.e., interview structuring, information gathering) for patient encounters [27]. Though meta-analyses have found CST to be effective, it focuses on a specific aspect of the patient encounter (one-on-one conversations), which is a small part of a resident’s overall clinical training [27].

### Need for clinical and situational context: situated learning theory

While the number of discrete clinical skills required for competent clinical practice may be overwhelming to trainees, organizing skills within a familiar context may overcome this challenge. Situated learning theory posits that mental schemas are formed based not only on what a learner commits to memory, but also on where and how the learning takes place (i.e., the physical and social environment, as well as the specific circumstance) [28]. Chess experts may have difficulty remembering strategies or piece positions from random boards, but they display uncanny precision when describing past matches [29]. Similarly, medical learners may struggle to learn from and practice a skill if they do not know the appropriate context for that skill and how subtle changes in a given situation may demand different actions.

As a result, the cognitive constructs trainees use to retrieve and practice skills need to be rooted not just in a *clinical *context (i.e., the medical circumstance in which an activity should be performed), but also in a *situational *context (i.e., location and time during a routine day in which the skill is utilized). If supervisors are not aligned with learners on how and where a skill should be demonstrated—both the *clinical *and *situational *contexts—feedback may suffer. As noted previously, milestones are context-independent and are meant to be applied in different settings. EPAs, on the other hand, are grounded in a *clinical* context but not in a specific *situational *context [30].

Consider how the skills within the pediatric EPA “*manage patients with acute, common diagnoses*” [2] can be demonstrated through different situational circumstances: patient presentations (e.g., expanding on a differential on rounds), bedside team huddles (e.g., escalating respiratory support based on expected illness trajectory), and family interactions (e.g., counseling families about testing options). Apart from referring to time/location circumstances, *situational *context can also delineate skills with additional descriptors: is this a presentation for a new patient or an existing patient that has been in the hospital for several days? Does the skill involve communication within the laboratory/imaging section of the presentation or the assessment section?

## Bringing microskill theory into the clinical setting

The creation of microskills in medicine is based on the premise that learner skill acquisition is directly related to personal comprehension and repeated engagement with a skill, feedback received, and the contextual environment in which one operates. Before designing a framework for clinical microskills, a comprehensive literature review of psychological microskills theory and effectiveness was performed, with a focus on meta-analyses of microcounseling [22, 31–33]. Several of these meta-analyses found statistically significant effect sizes for Kirkpatrick levels two (learning) and three (behavior), such as acquisition of microcounseling skills and improvement in social cognition [33]. However, some critics have questioned these meta-analyses, pointing to poor external validity (studies conducted in artificial environments), suboptimal dependent measures (e.g., patient self-reporting), and inadequate control groups [19]. Concurrently, these critics acknowledged microskills’ benefits in imparting basic skills and difficulties in proving educational technique effectiveness in randomized trials with real patients [19].

Additionally, while our own clinical experiences identified frustration in medical trainees’ experiences with current feedback frameworks, we reviewed the clinical coaching and feedback literature to identify a documented need for a new skills-based training methodology. Multiple analyses of trainees’ perceptions of current feedback systems show that trainees desire observations of specific actions with timely feedback on these tasks in a non-evaluative environment [34–36]. As one internal medicine resident stated in a focus group examining perceptions of feedback and the EPAs:

*I want* [feedback] *to be something similar to coaching in sports, or musical instruction where somebody who is an expert at a given skill actually observes … and can identify ways that* [you] *can improve doing that particular skill* [34].

Though trainees prefer targeted feedback on specific skills, current feedback tends to be generic and considered ineffective [34, 37]. In one study, only 56% of resident feedback focused on specific task practice, 13.7% contained action plans, and 3.9% described a performance gap [23]. Furthermore, when trainees perceive feedback to be evaluation-based, as opposed to coaching-based, they switch from a growth-mindset to a fixed-mindset, and stop attempting new skills that may invite poor evaluations [36, 38].

**Fig. 1:**
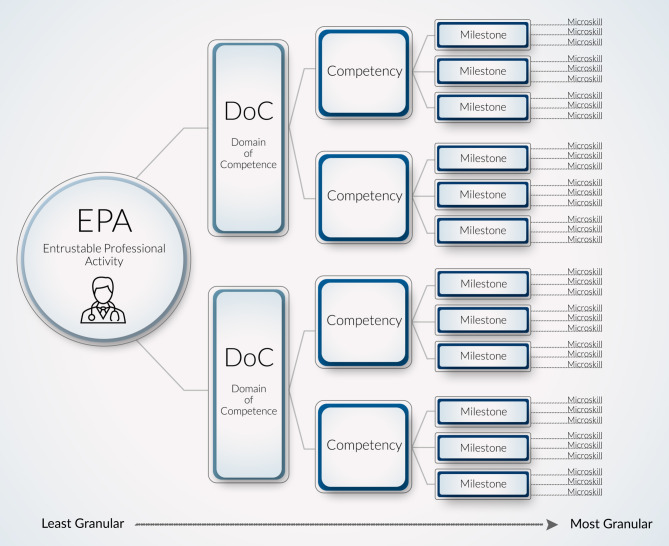
Relationship between entrustable professional activities (EPAs), milestones, and microskills. The EPAs are professional activities that define a profession and are comprised of one or more competencies, within domains of competence, and their associated milestones for development. By increasing granularity, which affords more specificity for feedback and improvement goals, microskills are broken down into situational and context-specific behaviors. Successfully mastering a broader EPA requires an attainment of advanced-level developmental competence and the completion of many smaller microskills

Given microskills effectiveness in other fields and opportunities for a skills-based feedback modality for medical trainees, we propose a set of clinical microskills, mapped to milestones and EPAs, for trainees to use on a daily basis as they develop skills in both clinical and situational contexts. Inspired by use in psychology and similar to how actions lend themselves to deliberate practice, each microskill is: *“(1) well-defined, (2) challenging yet achievable, (3) offers immediate feedback, (4) offers an opportunity to correct errors, and (5) offers an opportunity to repeat or practice until the skill becomes routine”* [39]. When creating microskills, we sought to define granular actions that, as a whole, combine to inform milestones, competencies, and EPAs (Fig. 1; see also Table S1 in the Electronic Supplementary Material [ESM]).

Microskills are distilled into small components and organized by the circumstances in which a learner would practice each action. They encompass several different areas; however, each one is targeted at the practices of an individual learner and built for deliberate practice. They are designed so an individual can choose a specific microskill and immediately know how to apply that skill in his/her day-to-day clinical routine. This learner-centered, context-based organization according to the daily components of a trainee’s workday differentiates microskills from EPAs (organized as a list of activities) and milestones (organized by competencies and domains of competence). Additionally, as microskills are structured to be clear and actionable, they allow for a shared language when soliciting and providing feedback. For example, if working on communication, does the learner use family-centered language in his/her assessments and address parental concerns naturally in each presentation? Similarly, an observer (e.g., senior resident, attending) can quickly review the chosen microskill and know where, when, and how the learner should apply that skill, thereby allowing him/her to provide targeted coaching.

Tab. 1 delineates the seven criteria for creating microskills. The microskill criteria incorporate action-based, granular components that allow for deliberate practice; applicable, observable, and learner-identified features that lend themselves to beneficial coaching; and a context-based organization derived from situated learning theory to mirror the learner’s workday.

**Table 1:** Microskill creation criteria. Each microskill was formed using the following criteria based on previously mentioned medical and negotiation theories of deliberate practice, coaching and formative feedback, as well as situated learning

*1. Action-based:* Microskills are written in active voice with action-based language. This format allows the trainee to recognize and undertake the desired skill, as opposed to leaving it up to interpretation as to how they should meet a goal
*2. Broadly-applicable yet narrowly-focused:* Microskills are vague enough that they can apply to multiple *types* of patients (i.e., patients with different diseases) yet narrow enough so that the trainee can identify the context in which to employ the skill (i.e., assessments for *existing *patients, as opposed to a *new *patient)
*3. Distilled into granular components:* Microskills are distilled into the most discrete components possible. For milestones/EPAs that mention various activities (*e.g., resuscitate, initiate stabilization of the acute patient*), each skill required of the trainee to fulfill that task is identified separately
*4. Organizationally designed according to the learner’s day:* The microskills are organized into domains, developed from a user-centered design approach that mirror a trainee’s workday
*5. Grounded in clinical and/or situational context:* Grouping microskills according to the circumstance in which they are employed helps to frame most microskills within a *situational *context. Depending on the microskill, some are also framed within a *clinical* context (i.e., the medical circumstance in which an activity should be performed)
*6. Easily observable:* The microskills contain enough descriptive context so they may be readily observed by supervisors, senior residents, and others to allow for feedback targeting precise skills
*7. Training-appropriate:* Microskills can be created and tailored for any training level and any specialty. The current set of microskills are designed for a pediatrics intern on a hospital medicine rotation, with the assumption that the learner has a working knowledge of physical exam skills and patient presentations from their medical school experiences. However, microskills can be created for any level of training

## Clinical microskill creation, features, and organization

### Considerations when creating clinical microskills

Microskills used for specific, actionable feedback targeting opportunities for continued growth can be developed for any specialty and any level of training. In order to demonstrate how microskills can be derived from existing assessment modalities and to emphasize the importance of incorporating user-centered design into the framework, we created a set of microskills for pediatric interns on a hospital medicine service using ACGME pediatric milestones and the American Board of Pediatrics’ (ABP) EPAs for general pediatrics [1, 2, 40]. Though we chose to use milestones and EPAs due to their widespread acceptance as assessment modalities, microskills can be derived from a variety of assessment frameworks (e.g., CanMEDS). To develop the microskills, the ACGME pediatric milestones, ABP EPAs, and ABP hospital medicine fellowship EPAs [41] were reviewed to determine those that were most appropriate for application in hospital medicine (Table S2, found in the ESM, lists the milestones and EPAs used for microskill development).

As we created microskills based upon our stated criteria, we found that some elements of existing assessment modalities were narrow enough to become microskills without significant adjustments, while others required more modifications. The EPA “*ensure complete handoffs using a standardized template when patients are transitioning from one care provider to another*” [2] served as a microskill almost verbatim, while the EPA “*synthesizing lab tests into proper patient care*” [2] was distilled into the ways an intern may engage with the skill (e.g., incorporating lab tests into presentations vs. written assessments vs. comparing patient labs with standards from an evidence-based literature review). Some EPAs required transformation from goals-based to observable skills-based actions. For example, an EPA focused on “*resuscitating *[the]* patient and then triage to align care with severity of illness*” [2] contains knowledge and management-based curricular components (*e.g., distinguishes between respiratory distress and failure*). However, a resident may struggle to explicitly apply this EPA to their day-to-day routine in an easily observable way. Distinguishing between respiratory distress and failure requires a specific context in which it can be applied, such as during a bedside presentation on rounds or through written notes that contain contingency planning.

As part of developing microskills mapped to milestones and EPAs, we created a user-centered framework focused on pediatric interns’ hospital medicine workday and the skills they use to navigate that day. User-centered design employs empathy to better understand the needs of the end-user and creates systems that complement the subject’s natural tendencies [42, 43]. By applying user-centered design to better understand the learner experience and through discussions with residents, we were able to imbue microskills with the relevant clinical and/or situational context, as well as construct a more naturally organized system for learners to select and practice skills. This process involved generating a user journey map—a tool used commonly in healthcare design to show how patients experience disease and interact with their care teams (see ESM, Fig. S1a) [44]. A user-centered skills map of *situational *context was also created based on the patient’s hospital experience and intern-patient interactions to demonstrate the possible skills a prototypical intern could employ at various stages of a patient’s hospitalization (see ESM, Fig. S1b). A *clinical *context map was not created, given that this context is already embedded in EPAs and would thus transfer easily onto the relevant microskills.

These user-centered maps were then combined with the extracted relevant milestones and EPAs to form situationally anchored microskills umbrellaed under a table of contents (see ESM, Fig. S2). The microskill table of contents mirrors the cognitive schema an intern uses to organize the workday, thereby lending itself to more natural microskill selection and adoption. The table of contents is an amalgamation of both the user-centered maps, incorporating unique aspects of the intern’s workday (e.g., pre-rounding, daily notes) as well as skills that surround the patient experience (e.g., delivering difficult news, family-focused presentations).

Groupings of skills that interns could situationally demonstrate to supervisors were separated into domains in the table of contents. For example, patient presentations are mentioned throughout multiple milestones [1] (*e.g., PC6: make informed diagnostic and therapeutic decisions that result in optimal clinical judgment*) and EPAs [2] (*e.g., EPA4: manage patients with acute, common diagnoses in an ambulatory, emergency, or inpatient setting*) and naturally align with both user maps. As a result, *patient presentations* became an independent microskill domain. However, even within *patient presentations*, an intern must employ different types of skills in various situational and clinical contexts, from integrating pathophysiology into everyday assessments, to creating contingency plans for acutely ill children, to relating to families in easy-to-understand language. Taking this into account, the categories within *patient presentations *were organized both by the features of presentations that the intern may be working on (the *situational* context—concise histories, tailored plans) as well as the types of patients discussed in the intern’s presentation (*clinical* context—new vs. multi-day vs. acutely ill patients).

An example of the many domains of competence (DoC), competencies, milestones, and microskills that inform a single EPA can be found in ESM, Fig. S3. (See ESM, Fig. S4 for an expanded example of the connections between entrustable professional activities (EPAs), milestones, and microskills).

### Clinical microskills in practice

While incorporation of microskills into practice will vary by institution, level of training, and clinical rotation, here we outline how a pediatric intern could use microskills on a hospital medicine rotation. When a new supervisor begins clinical service with the resident team, the intern would select one to three microskills (from the library of previously developed and disseminated microskills) to practice and be observed for the upcoming week. Though the intern will ultimately choose the skills he/she will practice, this should be a collaborative process with input from the supervisor and senior residents, considering skills the intern practiced in previous weeks. Throughout the week, both the intern and the supervisor know which skills are being practiced, thereby allowing the intern to refine the skills, as well as provide an opportunity for the supervisor to give real-time feedback on observations (or take notes to provide feedback at a later time). As one example, supervisors already accompany and observe trainees on clinical rounds, which enables a natural incorporation of the microskills framework. Supervisors can choose to deliver coaching in between trainees’ patient presentations or find time later in the day for more involved feedback. If supervisors find themselves pressed for time during or after rounds, they can consider integrating microskills feedback into their workflow later in the workday, such as by encouraging trainees to practice skills in follow-up conversations with patients.

Prior studies demonstrate that unless trainees have an expected forum to ask supervisors for feedback, they will instead forgo asking for feedback altogether [34]. Awareness by both the intern and supervisor of the chosen microskills at the beginning of the week increases the opportunity for feedback touchpoints and reduces the social anxiety trainees experience when asking for feedback out of context [34]. Additionally, when trainees know that the feedback they receive is based on direct observation, they are more likely to accept its accuracy and incorporate changes into practice [37]. At the end of the week, the intern, supervisor, and senior residents may review the intern’s chosen microskills, supervisor observations, and create action plans for intern skill improvement. This is also an opportunity for the supervisor to suggest new or repeat skills for the intern practice for the following week.

## Microskill challenges

Microskills have the potential to fill a current need in the medical training landscape, however, they are not without limitations and will benefit from further testing and refinement. Given the difficulty in demonstrating the effectiveness of randomized educational interventions, meta-analyses of microcounseling skills have come under recent criticism and it is unknown if previously demonstrated effectiveness will translate to medical training [19]. The microcounseling meta-analyses also note the lack of evidence for imparting complex skills and that distilling tasks into granular components may not prepare trainees for complicated real-life situations. However, existing skills-based clinical frameworks (e.g., CST, procedural training) have demonstrated trainees’ ability to learn complex skills (e.g., end-of-life counseling, surgical techniques) [26, 27, 45]. It remains unclear if trainees will more readily adopt some of our more basic microskills (e.g., performing physical exams) compared to the more complicated skills in our framework (e.g., leading acute patient care huddles).

The microskills framework may also become cumbersome if not created and implemented thoughtfully. The number of skills derived from milestones and EPAs can be numerous, and trainees need ways to sort through microskills to select those most relevant for their deliberate practice. Additionally, challenges may develop in standardizing how trainees communicate identified microskills with supervisors and how supervisors track observations and provide timely feedback.

Finally, selecting relevant skills requires deft supervisor coaching and a shared understanding that becoming competent in certain skills may not equate to achieving proficiency as a physician. Given their specificity, microskills are not built to offer holistic feedback to a training physician. Programs need a wide array of feedback tools to assist resident progress and microskills can add to, but should not replace, existing assessment methods.

## Conclusions and future directions

Microskills complement milestones and EPAs by making feedback and areas of focus for continued development more specific and actionable in clinical and situational contexts. Building on these strengths, they offer an opportunity to improve day-to-day learner feedback and bring granularity to the personal improvement process, based on deliberate practice and situated learning theory.

Future directions for microskills development include eliciting end-user perspectives on microskills compared to currently used assessment and feedback modalities as well as assessing microskill implementation outcomes. Future work should also focus on standardizing microskill implementation, creating microskills for other clinical contexts, specialties, and training levels, and developing the microskills coaching process for senior residents and supervisors. As microskills are created, each set needs to be refined by trainees, supervisors, and rotation directors.

## Supplementary Information

**Fig. S1** ***a, b*** User-centered design maps used to create and organize microskills. Maps depict the skills a prototypical intern might employ throughout the workday as well as how a hospitalized patient might interact with that intern

**Fig. S2 **Applying user-centered domains to create microskills categories: The microskill table of contents. The organization of the table of contents was created according to a user-centered approach, building off of designed user maps, in order to mirror the cognitive schemas a prototypical intern uses to organize the workday

**Fig. S3** An example of the connections between EPAs, milestones, and microskills. An example of the many domains of competence (DoC), competencies, milestones, and microskills that compose a single EPA. For a learner to successfully complete the EPA “*manage patients with acute, common diagnoses in an inpatient setting*”, they need to develop competency in patient care (*PC6: make informed diagnostic and therapeutic decisions…*) along various milestones. This involves first learning how to successfully navigate a myriad of microskills in different contexts (*e.g., use family-friendly language to explain complex concepts in patient presentations*; *differentiate between mild and severe symptoms that may point to impending deterioration*). The microskills in this example are only a few of the many possible options that compose each EPA and the subsequent milestones (see Fig. S4, also in ESM, for an expanded microskills example)

**Fig. S4** An expanded example of the connections between entrustable professional activities (EPAs), milestones, and microskills. Building off of the previous worked example in *Fig. S3 (also in ESM)*, this figure expands the many possible microskills that compose each EPA, competency, and subsequent milestones

**Table S1** Comparison of entrustable professional activities (EPAs), milestones, and microskills

**Table S2** List of milestones and entrustable professional activities (EPAs) included in microskills development
