# Supplementary material for: Building a doctor, one skill at a time: Rethinking clinical training through a new skills-based feedback modality
Source: Perspect Med Educ. 2021 May 26;10(5):304–11. doi: 10.1007/s40037-021-00666-9 (PMC8505598; doi:10.1007/s40037-021-00666-9)
Supplement: Supplementary file 1 — Fig. S1 a, b User-centered design maps used to create and organize microskills. Maps depict the skills a prototypical intern might employ throughout the workday as well as how a hospitalized patient might interact with that intern [file 40037_2021_666_MOESM1_ESM.docx]

| ***Fig. S1a,b*** User-centered design maps used to create and organize microskills  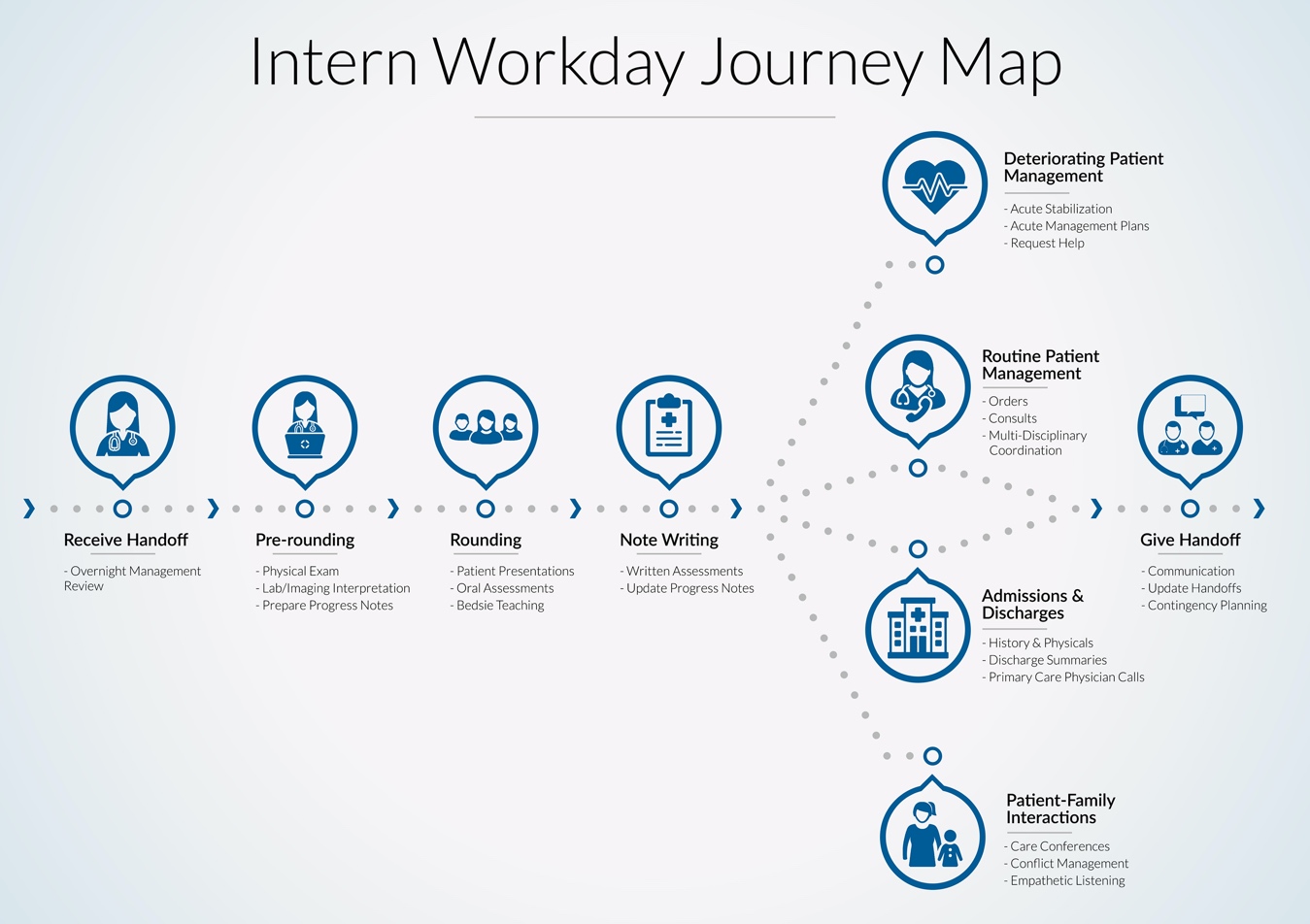  *Fig. S1a* A user-centered pediatric intern journey map, demonstrating how an intern goes through his/her workday. The intern’s workday is broken into points of significance, with each point representing a unique workday event or time-bound collection of tasks. Special workday events (*patient-family interactions* and *deteriorating patient management*) do not occur on a daily basis, however, were important enough to a prototypical intern’s cognitive construct (for how they classified skills) to merit inclusion. The journey map describes characteristic buckets of skills associated with each point in the workday, recognizing that some skills (*e.g.* physical exams) occur multiple times per day and could fall under several different time points (*e.g. pre-rounding*, *deteriorating patient management*). The skills associated with each workday event in the diagram are representative and not exhaustive  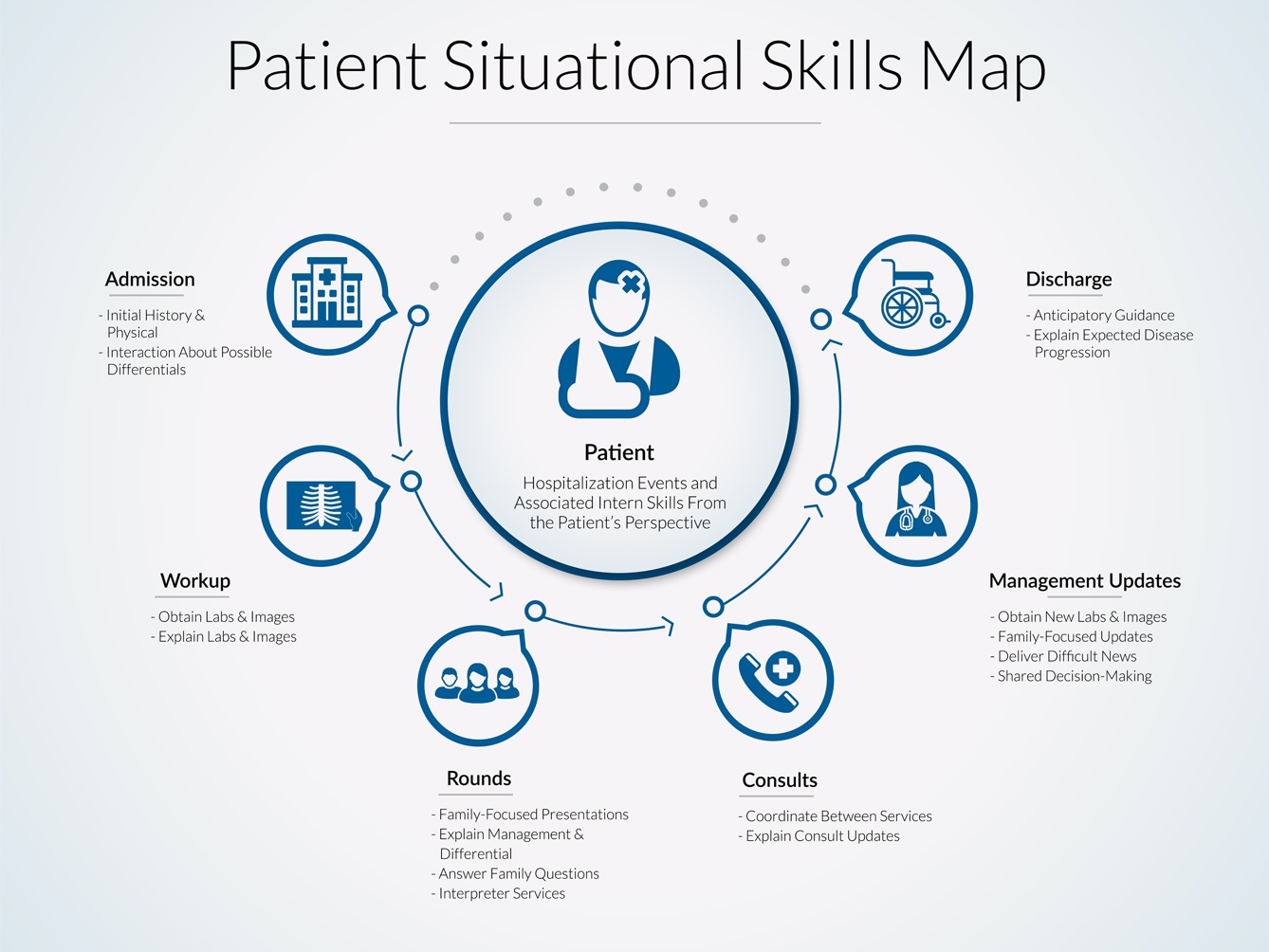  *Fig. S1b* A user-centered skills map demonstrating the hospitalization from the perspective of the patient, thereby highlighting situational events and patient-intern touchpoints. While this map has some similarities to the intern’s workday map, it is unique in that it is from the patient’s point of view and *only* concerns how the intern interacts with the patient and not the intern’s ‘behind-the-scenes’ work (*i.e.* note-writing would not be included). As a result, it points out new representative buckets of skills based on what the patient experiences, such as family communication on rounds or how the intern responds to labs/images throughout the patient’s workup |
| --- |
