# Supplementary material for: Building a doctor, one skill at a time: Rethinking clinical training through a new skills-based feedback modality
Source: Perspect Med Educ. 2021 May 26;10(5):304–11. doi: 10.1007/s40037-021-00666-9 (PMC8505598; doi:10.1007/s40037-021-00666-9)
Supplement: Supplementary file 2 — Fig. S2 Applying user-centered domains to create microskills categories: The microskill table of contents. The organization of the table of contents was created according to a user-centered approach, building off of designed user maps, in order to mirror the cognitive schemas a prototypical intern uses to organize the workday [file 40037_2021_666_MOESM2_ESM.docx]

| **Fig. S2** Applying user-centered domains to create microskills categories: The microskill table of contents. The organization of the table of contents was created according to a user-centered approach, building off of designed user maps, in order to mirror the cognitive schemas a prototypical intern uses to organize the workday  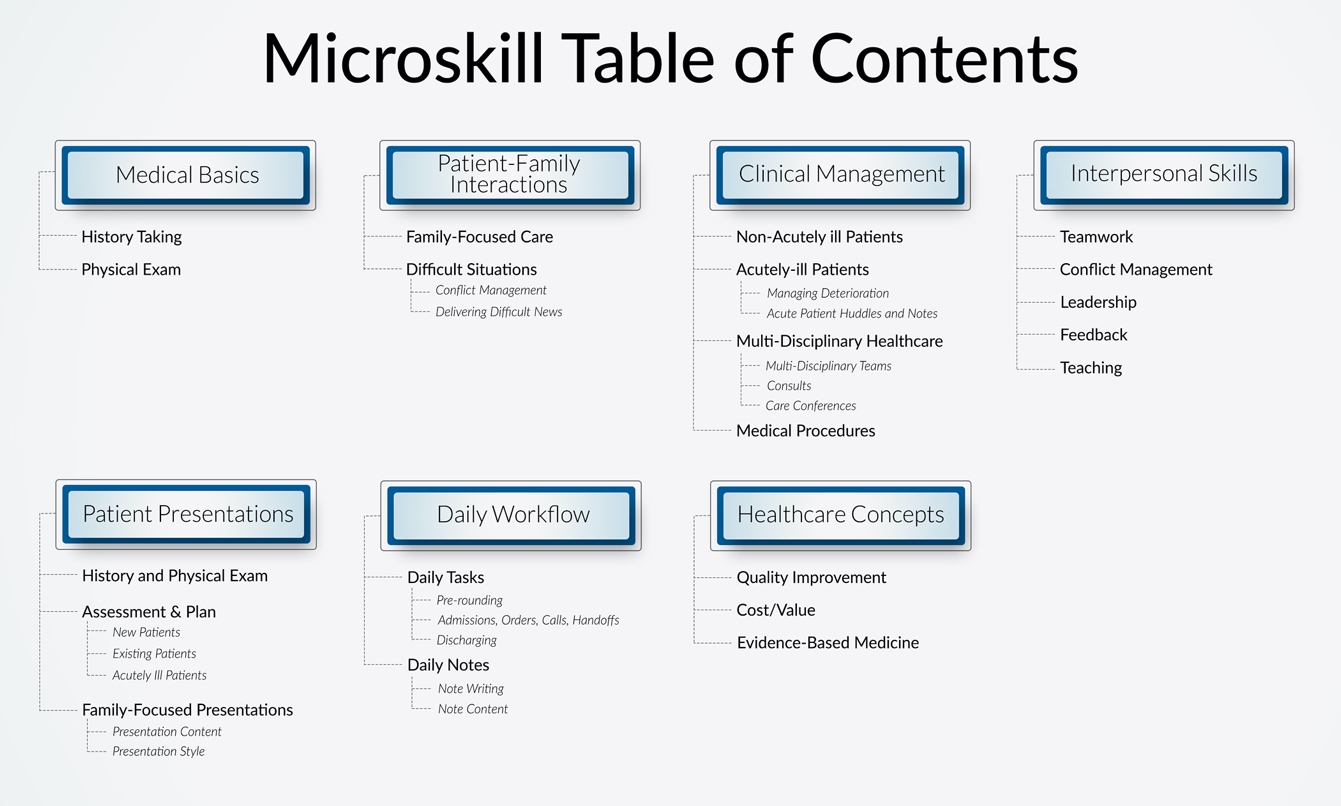 |
| --- |
