# Supplementary material for: Building a doctor, one skill at a time: Rethinking clinical training through a new skills-based feedback modality
Source: Perspect Med Educ. 2021 May 26;10(5):304–11. doi: 10.1007/s40037-021-00666-9 (PMC8505598; doi:10.1007/s40037-021-00666-9)
Supplement: Supplementary file 3 — Fig. S3 An example of the connections between EPAs, milestones, and microskills. An example of the many domains of competence (DoC), competencies, milestones, and microskills that compose a single EPA. For a learner to successfully complete the EPA “manage patients with acute, common diagnoses in an inpatient setting”, they need to develop competency in patient care (PC6: make informed diagnostic and therapeutic decisions…) along various milestones. This involves first learning how to successfully navigate a myriad of microskills in different contexts (e.g., use family-friendly language to explain complex concepts in patient presentations; differentiate between mild and severe symptoms that may point to impending deterioration). The microskills in this example are only a few of the many possible options that compose each EPA and the subsequent milestones (see Fig. S4, also in ESM, for an expanded microskills example) [file 40037_2021_666_MOESM3_ESM.docx]

| **Fig. S3** An example of the connections between EPAs, milestones, and microskills. An example of the many domains of competence (DoC), competencies, milestones, and microskills that compose a single EPA. For a learner to successfully complete the EPA “*manage patients with acute, common diagnoses in an inpatient setting*”, they need to develop competency in patient care (*PC6: make informed diagnostic and therapeutic decisions…*) along various milestones. This involves first learning how to successfully navigate a myriad of microskills in different contexts (*e.g.,* *use family-friendly language to explain complex concepts in patient presentations*; *differentiate between mild and severe symptoms that may point to impending deterioration*). The microskills in this example are only a few of the many possible options that compose each EPA and the subsequent milestones (see Fig. S4, also in ESM, for an expanded microskills example)  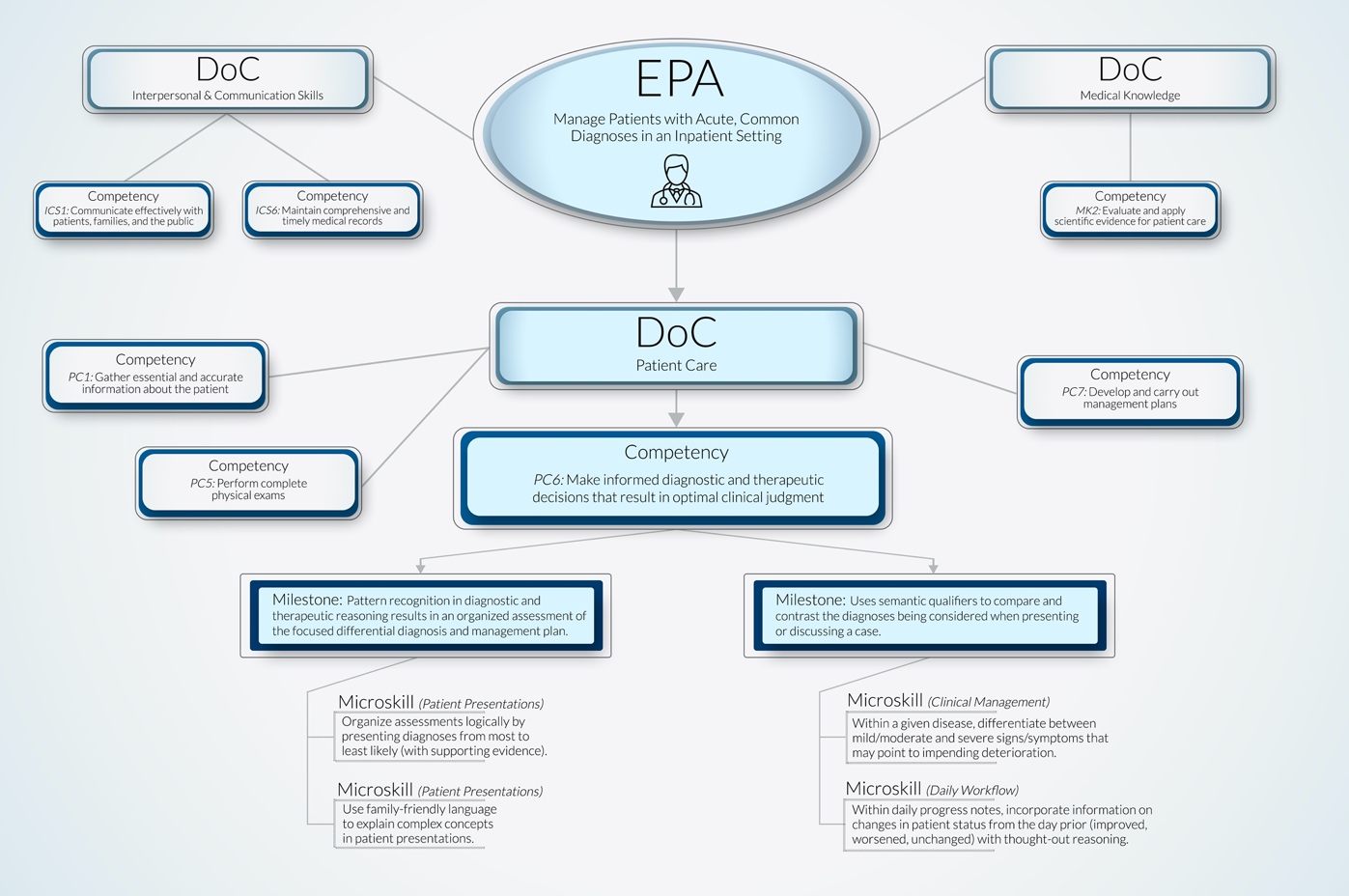 |
| --- |
