# Supplementary material for: Building a doctor, one skill at a time: Rethinking clinical training through a new skills-based feedback modality
Source: Perspect Med Educ. 2021 May 26;10(5):304–11. doi: 10.1007/s40037-021-00666-9 (PMC8505598; doi:10.1007/s40037-021-00666-9)
Supplement: Supplementary file 4 — Fig. S4 An expanded example of the connections between entrustable professional activities (EPAs), milestones, and microskills. Building off of the previous worked example in Fig. S3 (also in ESM), this figure expands the many possible microskills that compose each EPA, competency, and subsequent milestones [file 40037_2021_666_MOESM4_ESM.docx]

| **Fig. S4** An expanded example of the connections between entrustable professional activities (EPAs), milestones, and microskills. Building off of the previous worked example in *Fig. S3 (also in ESM)*, this figure expands the many possible microskills that compose each EPA, competency, and subsequent milestones  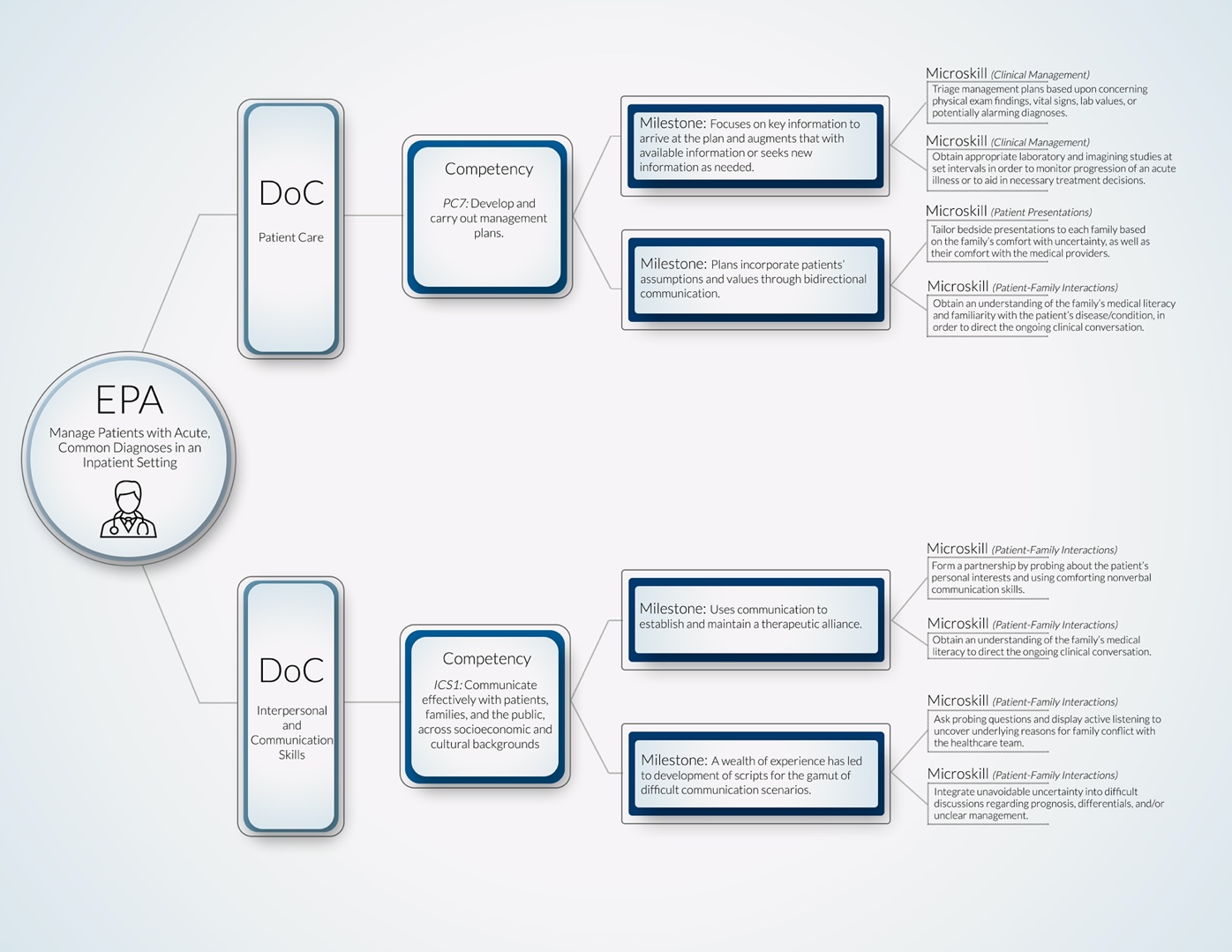 |
| --- |
