# Supplementary material for: Building a doctor, one skill at a time: Rethinking clinical training through a new skills-based feedback modality
Source: Perspect Med Educ. 2021 May 26;10(5):304–11. doi: 10.1007/s40037-021-00666-9 (PMC8505598; doi:10.1007/s40037-021-00666-9)
Supplement: Supplementary file 5 — Table S1 Comparison of entrustable professional activities (EPAs), milestones, and microskills [file 40037_2021_666_MOESM5_ESM.docx]

**Table S1** Comparison of entrustable professional activities (EPAs), milestones, and microskills

|  | *What is it?* | *Is it granular?* | *Is it observable?* | *How can it be used for trainee assessments?* | *How is the content organized?* | *Does it incorporate clinical and situational context?* |
| --- | --- | --- | --- | --- | --- | --- |
| ***EPAs*** | List of activities that compose the professional scope of practice for a specialty | No, EPAs are synthetic and performing each one requires incorporation of many competencies, milestones, and microskills | Yes, EPAs are holistic observable activities | Useful for holistic assessments; entrustment decisions based on trainee performance of an activity | List of activities that define the professional scope of practice for a specialty | Grounded in clinical context; situational context independent |
| ***ACGME Milestones*** | Narrative descriptions of development within competencies | Sometimes, depending on the competency / milestone | Sometimes, depending on the competency/milestone | Useful for assessments targeted in competency areas; places trainees along a continuum of development | Organized by competencies and their larger domains of competence | Clinical and situational context independent |
| ***Microskills*** | Granular action-based skills | Yes, microskills are discrete actions | Yes, microskills are easily observable actions | Useful for day-to-day assessments of specific actions; not intended for holistic trainee assessments | Organized by a learner-centered context and where/how skills are employed within the workday | Grounded in clinical and situational context |
