# Supplementary material for: Building a doctor, one skill at a time: Rethinking clinical training through a new skills-based feedback modality
Source: Perspect Med Educ. 2021 May 26;10(5):304–11. doi: 10.1007/s40037-021-00666-9 (PMC8505598; doi:10.1007/s40037-021-00666-9)
Supplement: Supplementary file 6 — Table S2 List of milestones and entrustable professional activities (EPAs) included in microskills development [file 40037_2021_666_MOESM6_ESM.docx]

**Table S2** List of milestones and entrustable professional activities (EPAs) included in microskills development

| Microskills were derived most heavily from the following ACGME Pediatric Competencies:   - *PC1:* Gather essential and accurate information about the patient - *PC2:* Organize and prioritize responsibilities to provide patient care that is safe, effective, and efficient - *PC4:* Interview patients/families about the particulars of the medical condition for which they seek care, with specific attention to behavioral, psychosocial, environmental, and family-unit correlates of disease - *PC6:* Make informed diagnostic and therapeutic decisions that result in optimal clinical judgment - *PC7:* Develop and carry out management plans - *PC9:* Counsel patients and families - *PC10:* Provide effective health maintenance and anticipatory guidance - *MK1:* Demonstrate sufficient knowledge of the basic and clinically supportive sciences appropriate to pediatrics - *PBLI9:* Participate in the education of patients, families, students, residents, and other health professionals - *ICS1:* Communicate effectively with patients, families, and the public, as appropriate, across a broad range of socioeconomic and cultural backgrounds - *ICS2:* Demonstrate the insight and understanding into emotion and human response to emotion that allow one to appropriately develop and manage human interactions - *ICS3:* Communicate effectively with physicians, other health professionals, and health-related agencies - *ICS4:* Work effectively as a member or leader of a health care team or other professional group - *ICS6:* Maintain comprehensive, timely, and legible medical records, if applicable - *SBP2:* Coordinate patient care within the health system relevant to their clinical specialty - *PPD4:* Practice flexibility and maturity in adjusting to change with the capacity to alter behavior - *PPD8:* Recognize that ambiguity is part of clinical medicine and respond by utilizing appropriate resources in dealing with uncertainty   Microskills were derived most heavily from the following ABP EPAs:   - *EPA 4:* Manage patients with acute, common diagnoses in an ambulatory, emergency, or inpatient setting - *EPA 5:* Provide a medical home for well children of all ages - *EPA 10:* Resuscitate, initiate stabilization of the patient and then triage to align care with severity of illness - *EPA 11:* Manage information from a variety of sources for both learning and application to patient care - *EPA 15:* Lead an interprofessional health care team - *Pediatric HM EPA:* Direct and coordinate care for patients with unclear diagnoses and complex patients - *Pediatric HM EPA:* Teach, provide feedback, and assess learners across a competency-based medical education continuum and engage in interprofessional education |
| --- |
